# Œsophageal Obstruction

**Published:** 1901-06

**Authors:** F. R. Sanders

**Affiliations:** Corydon, Iowa


### ŒSOPHAGEAL OBSTRUCTION.

By F. R. Sanders, M.D.C.,
CORYDON, IOWA.

When œsophageal obstruction is due to farinaceous matter and cannot be moved by manipulation, I get satisfactory results by simply forcing water into the gullet. The *modus operandi* is very easy, one will perceive, especially when urged by necessity. However, I have had two cases which demanded additional treatment, a brief report of which may be of interest.

Case I.—Brown mare, draught, seven years old, in good condition. I found the animal suffering from low cervical choke of four hours' standing. I administered a dose of oil and applied digital manipulations, but failed to move the offending material. I procured eight feet of rubber hose and attempted several times to pass it in the œsophagus down to the bolus, but I was compelled to abandon it for the time being, as respiration would completely cease when the hose entered the gullet, presumably because it interfered with the action of the epiglottis. I immediately performed tracheotomy, after which the hose was gently passed down until it came in contact with the bolus. An injection pump was attached. The muscles were compressed around the œsophagus upon the hose by the hands, with the view of preventing the return of water. We next pumped in about two and a half gallons of water. The hose was now withdrawn, and the skin sutured over the seat of tracheotomy. The animal was able to work the next day, and made a complete recovery.

CASE II.—Bay mare, six years old, roadster, in very good flesh. Was called the evening of September 18, 1900, but I was not able to see the patient until September 19th at 9 A.M. The owner said that the animal became choked while eating oats at noon on September 18th; that he tried different ways to relieve her, and, finally, acting upon someone's advice, as a cure, he hitched her to the plough and worked her the remainder of the day. This animal at the time of operation had a temperature of 104° F., labored breathing, and showed much pain when attempting to swallow. I decided the choke was thoracic, and proceeded as in Case I. (excepting oil and massage, of course), but I could not apply sufficient pressure to prevent the water from returning and endangering the entrance of foreign substances into the larynx. Knowing the gravity of the situation, I decided to make an incision about four inches long through the skin parallel with the œsophagus and between the muscles until I was able to grasp the œsophagus with my left hand, allowing the fingers to encircle it. While holding it in this position an assistant applied the pump until there was little or no resistance. After withdrawing the hose the animal was blanketed and returned to the stall. The wound in the neck was not sutured, and antiseptic measures were not taken. I instructed the owner to give occasional small doses of olive oil for two or three days. Very little nourishment was taken the first two days after the operation. The mare recovered completely after the lapse of one week, except a slight cough, which disappeared a week later.

